# Predicting resection success in giant pituitary adenomas: morphologic determinants and a preoperative multivariate model

**DOI:** 10.3389/fendo.2026.1759071

**Published:** 2026-01-30

**Authors:** Dilan Ozaydin, Ender Vergili, Alperen Kaya, Ahmet Numan Demir, Pinar Kadioglu, Necmettin Tanriover

**Affiliations:** 1 Department of Neurosurgery, Stanford University, Palo Alto, CA, United States; 2 Department of Neurosurgery, Istanbul University-Cerrahpaşa, Istanbul, Türkiye; 3 Department of Endocrinology, Metabolism, and Diabetes, Istanbul University-Cerrahpasa, Istanbul, Türkiye

**Keywords:** cranial extension, endoscopic transsphenoidal surgery, giant pituitary adenoma, multilobulated adenoma, resection predictors, tumor morphology

## Abstract

**Introduction:**

Giant pituitary adenomas present significant therapeutic challenges due to their size, complex morphology, and frequent extension into critical neuroanatomical regions. Endoscopic transsphenoidal surgery (ETSS) has become the preferred first-line treatment, yet factors determining resectability remain incompletely defined. This study retrospectively analyzed 60 patients with giant pituitary adenomas treated via ETSS at a tertiary pituitary center to evaluate clinical characteristics, early surgical outcomes, and preoperative determinants of resection. Visual impairment was the most common presenting symptom and improved in 83.7% of patients. Based on early postoperative imaging, total or gross-total resection was achieved in 76.7% of the cohort, and permanent diabetes insipidus occurred in only 3.3%, with no major vascular or neurological complications observed. Larger tumor volume and maximum diameter, multilobulated morphology, cranial extension pattern, and higher Hardy grades were independently associated with limited resection. A multivariate model integrating volumetric, anatomical, and hormonal variables demonstrated moderate predictive accuracy for identifying patients at risk for subtotal resection (AUC = 0.745). These findings highlight the critical role of tumor geometry and extension patterns in determining the feasibility of complete removal and provide a practical framework for preoperative risk stratification. ETSS provides effective early tumor debulking in patients with giant pituitary adenomas; however, longer-term follow-up is required to fully assess the durability of surgical outcomes and disease control.

**Methods:**

We retrospectively analyzed 60 consecutive patients with giant pituitary adenomas who underwent endoscopic transsphenoidal surgery (ETSS) at a tertiary pituitary center between 2015 and 2023. Clinical, radiological, hormonal, and surgical variables were collected from medical records. Tumor volume, cranial extension pattern, Hardy and Knosp classifications, and multilobulated morphology were evaluated using preoperative MRI. Surgical outcomes, complications, and postoperative endocrine and visual results were recorded. Predictors of resection extent were assessed using multivariate logistic regression, and a preoperative prediction model was developed and evaluated with ROC analysis.

## 1 Introduction

Pituitary adenomas are intracranial neoplasms that arise from the pituitary gland and account for approximately 10-15% of all primary brain tumors (1). While most pituitary adenomas are small and have a benign course, treating giant pituitary adenomas can be challenging (2). Giant pituitary adenomas, defined as tumors larger than 4 cm in diameter, are relatively rare but can have a significant impact on patients in terms of morbidity and mortality (3). Effective diagnosis and treatment of giant pituitary adenomas requires a comprehensive understanding of their clinical features, radiologic characteristics, and treatment options (4). These tumors exhibit a peculiar behavior, often showing more aggressive growth and causing widespread effects on surrounding structures, leading to various clinical symptoms and complications. In addition, the clinical picture is further complicated by the potential for hormonal dysregulation (3, 4). Treating giant pituitary adenomas requires a multidisciplinary approach involving neurosurgeons, endocrinologists, radiation oncologists, and other specialists (5, 6). The available treatment options include surgical resection, drug therapies, and radiotherapy. However, the optimal treatment strategy is influenced by various factors, including tumor size, location, invasiveness, and the patient’s overall health (2–4). Except for prolactinomas, surgery is generally considered the first-line treatment for most large pituitary tumors. The primary goal is to achieve the safest possible resection while preserving neurological function. Commonly used procedures include microscopic transsphenoidal surgery and transcranial procedures (7, 8). With the advent of endonasal endoscopic transsphenoidal surgery (EETS) in the late 1990s, the scope of transnasal procedures has expanded to include these tumors and the entire skull base (9). Currently, EETS is considered the primary surgical option for most giant pituitary adenomas in most referral centers. At the same time, the transcranial approach is reserved for selected cases with supra- and parasellar extensions (10).

Despite the growing body of literature on giant pituitary adenomas treated with EETS, several clinically relevant questions remain incompletely answered. Large single- and multicenter series have reported global outcomes and complication profiles, yet the specific impact of tumor morphology (e.g. multilobulated configuration) and detailed cranial extension patterns on resectability has not been systematically quantified. Moreover, the combined predictive value of volumetric parameters, anatomical classification systems, and the degree of anterior pituitary hormone deficiency for anticipating the extent of resection has been only partially explored. As a result, preoperative counseling and surgical planning in patients with giant adenomas often still rely on subjective estimation rather than on integrated, data-driven risk stratification.

This study presents data on the clinical features, diagnostic approaches, treatment strategies, and outcomes of giant pituitary adenomas treated at our pituitary center, where all patients underwent endoscopic transsphenoidal surgery (ETSS). In contrast to previous reports, we specifically evaluate how multilobulated morphology, cranial extension patterns, and endocrine status influence the extent of resection, and we develop a multivariate preoperative prediction model for total tumor removal. By focusing on a homogenous cohort operated on by a single experienced endoscopic neurosurgeon, we aim to elucidate anatomical and hormonal predictors that can guide surgical decision-making, inform the need for staged or adjunctive therapies, and refine patient counseling regarding expected surgical outcomes.

## 2 Materials and methods

### 2.1 Patients and data collection

This retrospective study was conducted at the Pituitary Centre of a tertiary care university hospital. The medical records of patients who underwent pituitary adenoma surgery at our center between 2015 and 2023 were retrospectively reviewed. The inclusion criteria were: (i) patients with pituitary adenomas with the largest diameter greater than 4 cm, regardless of hormone secretion; (ii) patients aged 18 years or older. Patients with incomplete information on the following clinical, biochemical, radiologic, and histopathologic features were excluded: (i) demographic characteristics (gender, age at diagnosis and surgery); (ii) radiological characteristics (tumor size at diagnosis based on maximum diameter in millimeters, presence of cavernous sinus invasion); (iii) biochemical results (assessment of pituitary hormones at diagnosis and during follow-up); (iv) immunohistochemical staining of pathological samples for hormone assessment.

Data were extracted from patient records for the study, including age at diagnosis, gender, presenting symptoms, pituitary adenoma size and volume, cranial extent, presence of cavernous invasion, pituitary hormone level assessment, preoperative and postoperative medication use, extent of surgical resection, pathology results, postoperative complications, presence of surgical remission, and follow-up information. The mortality data were taken from the government’s medical database, accessed at https://obs.saglik.gov.tr/Account/Login.

### 2.2 Endocrinological evaluation

The endocrinological parameters were evaluated according to internationally recognized guidelines, and the diagnosis of functioning pituitary adenomas was established according to standard clinical and biochemical criteria, in line with current international guidelines (11–18). Acromegaly was diagnosed based on elevated age- and sex-adjusted insulin-like growth factor 1 (IGF-1) levels and the absence of growth hormone (GH) suppression during oral glucose tolerance testing (12). Cushing’s disease was diagnosed in patients with clinical features of hypercortisolism accompanied by abnormal cortisol suppression testing and elevated adrenocorticotropic hormone (ACTH) levels (13, 14). Prolactinomas were diagnosed in the presence of markedly elevated serum prolactin concentrations after exclusion of secondary causes of hyperprolactinemia (11).

Fasting blood samples were taken from the participants between 8 and 9 am for the measurement of biochemical parameters, including ACTH, basal cortisol, thyroid stimulating hormone (TSH), free triiodothyronine (FT3), free thyroxine (FT4), GH, IGF-1, prolactin, follicle-stimulating hormone (FSH), luteinizing hormone (LH), total testosterone in men and estradiol in women. In cases where hormone deficiency was detected, appropriate hormone replacement therapy was administered according to current guideline recommendations (19).

### 2.3 Radiological evaluation

Before ETSS, all patients underwent high-resolution magnetic resonance imaging (MRI) of the pituitary and hypothalamus region. Until 2018, 1.5T MRI devices were used; after that, 3T MRI devices were used. The presence of a hypointense lesion after intravenous administration of gadolinium indicates the presence of a pituitary adenoma. The latero-lateral, dorsoventral and craniocaudal diameters of the adenomas were determined. Adenomas with a maximum diameter of≥ 40 mm were classified as giant adenomas. Images were examined, and suprasellar extension and cavernous sinus invasion were documented.

Cavernous sinus invasion was present when the adenoma extended beyond the lateral intercarotid line (20). The suprasellar extension was defined as the extension of the pituitary adenoma beyond the line drawn from the sella tuberosity to the posterior clinoid process in the sagittal/parasagittal plane (20, 21). The direction of the suprasellar extension was recorded as frontal, temporal, or towards the third ventricle. Tumor morphology was categorized on preoperative MRI as round, dumbbell-shaped, or multilobulated (22). A multilobulated adenoma was defined as a lesion demonstrating two or more distinct lobular components separated by visible constrictions or internal septations on MRI (22). All imaging-based classifications were independently assessed by two experienced neurosurgeons blinded to surgical outcomes, and discrepancies were resolved by consensus. Formal inter-rater reliability statistics were not calculated; however, all discrepant cases were jointly reviewed to reach a final consensus classification. The Hardy-Wilson classification was used to assess sellar destruction (grade) and extrasellar extension (stage) (20). The cavernous sinus invasion was evaluated using the Knosp-Steiner classification based on coronal T1-weighted contrast-enhanced imaging (21) and was corroborated by intraoperative findings.

To quantify tumor size, the maximum diameter of the tumor was measured in axial, sagittal, and coronal images using T1-weighted coronal slices with and without contrast enhancement. The tumor volume was calculated using the following formula: V = xyzπ/6 (where x is the length, y is the width, and z is the height).

### 2.4 Visual field evaluation

All patients were examined for visual field defects using the Humphrey visual field test preoperatively and during follow-up care.

### 2.5 Surgical technique and follow-up

All patients who underwent primary ETSS were operated on by a single senior neurosurgeon (N.T.) with over 25 years of experience in pituitary surgery, who had performed more than 800 endoscopic endonasal pituitary procedures during the study period. The surgical technique included a binostril approach with a broad anterior sphenoidotomy and a generous opening of the sella floor and dura in all cases. Given that all tumors included in the study were giant pituitary adenomas, extended endoscopic endonasal approaches, including transtubercular and transplanar modifications, were systematically employed. Intraoperative cerebrospinal fluid (CSF) leaks were repaired with autologous fat, muscle grafts, and the nasoseptal flap.

In the early postoperative period, patients were monitored for complications such as diabetes insipidus (DI) and hyponatremia due to inappropriate antidiuretic hormone secretion. All patients underwent an MRI of the sella within the first 24 hours after surgery. After discharge, patients were followed up at 3- and 6-month intervals during the first year after surgery. The follow-up examinations included a clinical assessment, MRI of the sella, measurement of hormone levels, and visual field tests.

To assess the extent of tumor resection achieved during surgery, an MRI of the sella was performed in the third postoperative month. The degree of tumor resection was defined as follows: total resection (100%), gross total resection (90-100%), and subtotal resection (< 90%) (23).

### 2.6 Histopathologic evaluation

Surgical specimens were collected for histopathologic analysis, which included standard hematoxylin and eosin staining, periodic acid–Schiff staining, and reticulin staining. In addition, immunohistochemical staining was performed to determine pituitary hormones, Ki67 expression, and other relevant markers. The Ki67 score, which represents the proliferation index, and the presence of mitotic activity were reported as part of the histopathologic findings.

### 2.7 Assays

Serum levels of ACTH, basal cortisol, TSH, FT3, FT4, GH, IGF-1, prolactin, FSH, LH, total testosterone, and estradiol were measured using an electrochemiluminescence immunoassay (ECLIA) on a Roche Cobas e system (Roche, Cobas e 602, Roche Diagnostics GmbH, Mannheim, Germany).

### 2.8 Statistical analysis

The statistical analyses in this study were carried out using the Statistical Package for the Social Sciences (SPSS) software, in particular version 27.0. The normality of the data was tested using the Kolmogorov-Smirnov test. Continuous variables were expressed as mean ± standard deviation (SD) or median with interquartile range (IQR) if the data were not normally distributed. Student’s t-tests or analyses of variance (ANOVA) were used to compare means between groups when data were normally distributed. In cases where the data did not meet the normality assumption, the Mann-Whitney U test or Kruskal-Wallis test was used to compare medians. The correlation coefficients between continuous variables were calculated using either the Spearman rank-order or Pearson correlation tests. The frequencies were compared using Pearson’s chi-square or Fisher’s exact tests. The significance level was set at p < 0.05, and all results were interpreted with a 95% confidence interval.

## 3 Results

### 3.1 General characteristics

Between 2015 and 2023, our center operated on 700 patients with pituitary adenomas. The study included 60 patients with giant pituitary adenomas who underwent 72 ETSS. **Table 1** contains the general characteristics of the patients. Of the patients, 40 (66.6%) were male; the mean age was 49 ± 13.2 years. The most common complaint of the patients was visual impairment. **Table 2** shows the results of the preoperative hormone testing, which revealed that 49 patients (81.7%) had at least one anterior pituitary hormone deficiency before surgery.

**Table 1:** General characteristics of patients.

Characteristics	Number of patients	Percentage (%)
Presenting symptoms		
Headache	22	(36.7)
Visual disturbances	47	(78.3)
Constitutional	14	(23.3)
Oligomenorrhea/loss of libido	12	(20)
Cranial nerve palsy	4	(6.7)
Preoperative visual field deficit	49	(81.7)
Shape of adenoma		
Round	22	(36.7)
Dumbbells	6	(10)
Multilobulated	32	(53.3)
Cranial extension		
Absent	32	(53.3)
Frontal lobe	12	(20)
Temporal lobe	8	(13.3)
Third ventricle	12	(20)
Cavernous sinus invasion	58	(96.7)
Unilateral	12	(20)
Bilateral	46	(76.7)

**Table 2:** Preoperative hormonal evaluation of patients.

Preoperative hormonal evaluation	Number of patients	Percentage (%)
Clinically non-functioning	38	(63.3)
Clinically functioning	22	(36.7)
Prolactinoma	12	(20)
Acromegaly	8	(13.3)
Cushing Disease	2	(3.3)
Number of anterior pituitary hormones in failure		
0	11	(18.3)
1	15	(25)
2	18	(30)
3	10	(16.7)
4	6	(10)
Type of anterior pituitary hormones in failure		
Adrenocorticotropic hormone (ACTH)	14	(23.3)
Gonadotropin hormones (GnH)	32	(53.3)
Growth hormone (GH)	23	(38.3)
Thyroid-stimulating hormone (TSH)	36	(60)

### 3.2 Radiological features

**Figure 1:**
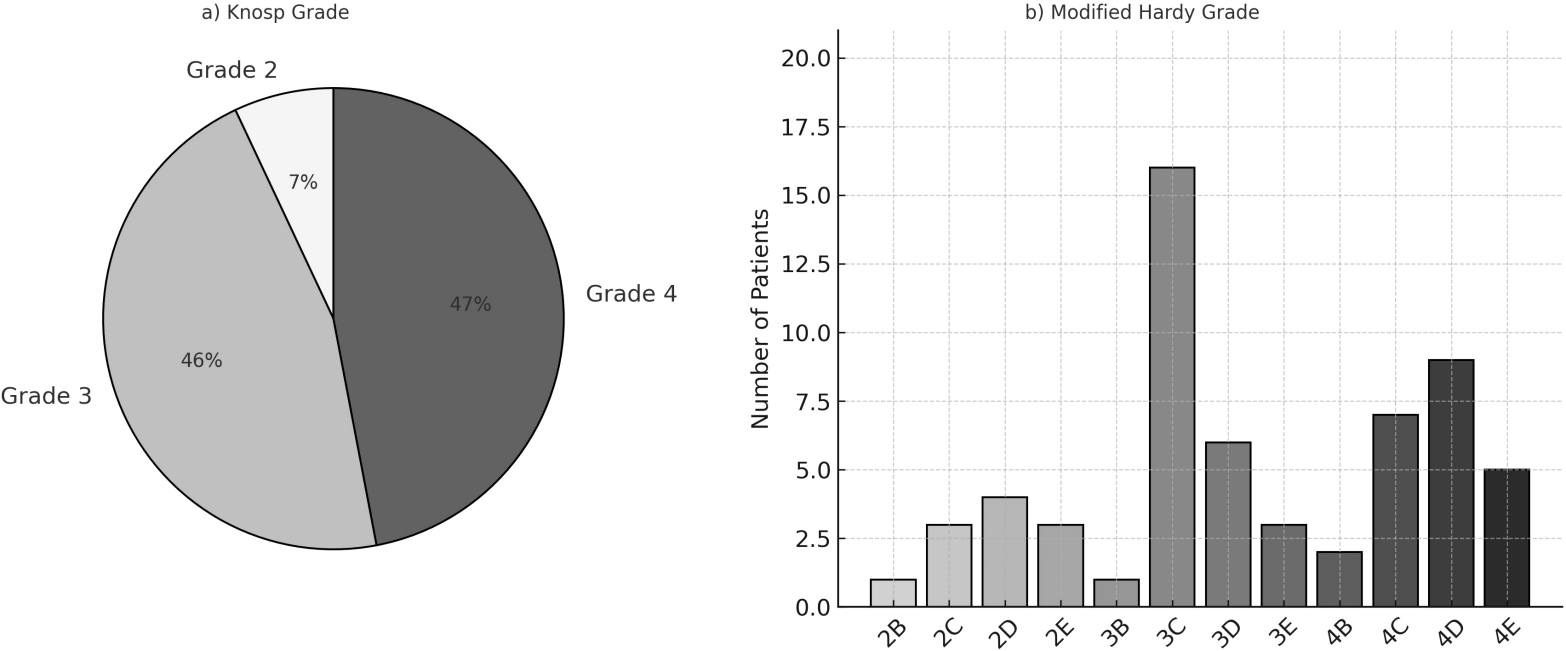
**(a)** Preoperative Knosp grade of patients’ pituitary adenomas. **(b)** Preoperative Modified Hardy grade of patients’ pituitary adenomas.

**Figure 2:**
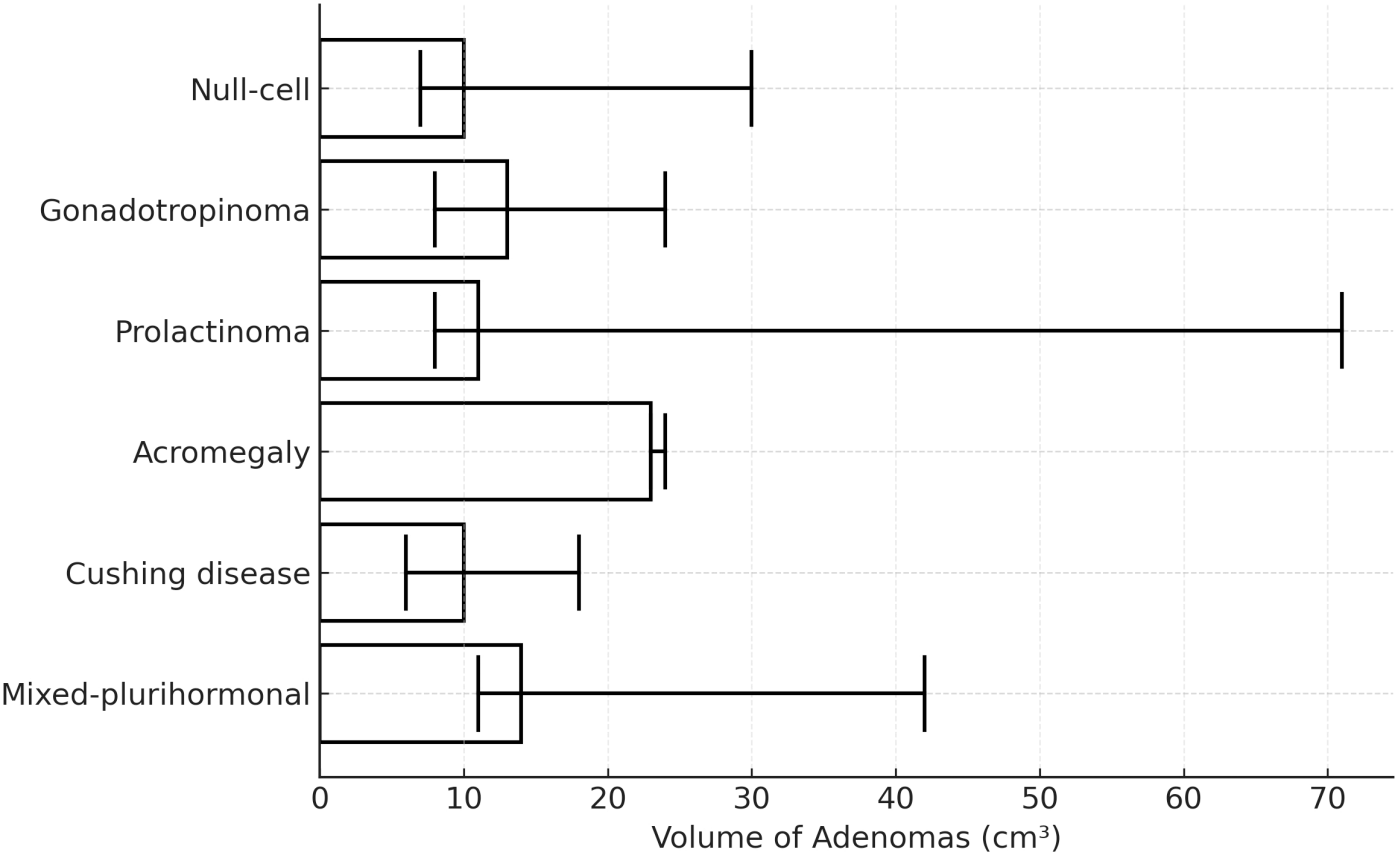
Comparison of median adenoma volumes according to pathology results. The median volume of null-cell adenomas was 10 cm3 [13 – 30]; the median volume of gonadotropin-secreting adenomas was 13 cm3 [18 – 24]; the median volume of prolactin-secreting adenoma was 11 cm3 [14 – 71]; the median volume of somatotropin-secreting adenoma was 23 cm3 (24); the median volume of corticotropin-secreting adenoma was 10 cm3 [14–18]; the median volume of mixed-plurihormone-secreting adenomas was 14 cm3 [17–42] (p = 0.956).

The maximum diameter of the adenomas was 44.8 ± 5.4 mm, and the median volume was 17.3 [12–24.5] cm^3^. Of the patients, 28 (46.7%) had a cranial extension of the pituitary adenomas. **Table 1** and **Figure 1** show the preoperative radiologic features. **Figure 2** compares median adenoma volumes based on pathology results, and no significant difference was found between adenoma volumes based on pathology results (p = 0.956).

### 3.3 Surgical results

As shown in **Table 3**, total resection was achieved in 26 patients (43.3%). There were no cases of cerebrospinal fluid (CSF) loss, meningitis, vascular complications, or operation-related deaths in the postoperative period. Permanent diabetes insipidus was only observed in 2 patients (3.3%). In 6 patients (10%), the hormone deficiency improved in the postoperative phase. Postoperative visual field defects were completely or partially resolved in 41 patients (83.7%). The pathology results are presented in **Table 3** and show a mean number of mitoses per 10 high-power fields of 1.05 ± 0.97 and a median Ki-67 labeling index of 3 (2–4) %.

**Table 3:** The operation, pathology, and follow-up results.

Postoperative results	Number of patients	Percentage (%)
Adenoma hormone staining results		
Null-cell adenoma	3	(5)
Gonadotropin adenoma	32	(53.3)
Prolactin-secreting adenoma	11	(18.3)
Growth hormone-secreting adenomas	2	(3.3)
ACTH-secreting adenoma	2	(3.3)
Mixt-plurihormonal adenoma	10	(16.7)
Amount of resection		
Total resection	26	(43.3)
Gross-total resection	20	(33.3)
Subtotal resection	14	(23.4)
Recovery in the field of vision	41	(83.7)
Persistent diabetes insipidus	2	(3.3)
Recurrence	12	(20)
Recovery from the hormone deficiency	6	(10)

### 3.4 Follow-up results

The patients were followed up over 31.3 ± 18 months. During this period, the ETSS was repeated in 12 patients. Indications for repeat ETSS included recurrence of an adenoma and removal of a residual pituitary adenoma that had collapsed into the sella after subtotal resection. In the postoperative phase, eight patients (13%) with prolactinoma received dopamine agonists, six patients (10%) with acromegaly were treated with somatostatin receptor ligands, and one patient (1.7%) with Cushing’s syndrome received both a dopamine agonist and a somatostatin receptor ligand, pasireotide. Four patients (6.7%) received gamma knife treatment. Two patients (3.3%) had a malignant prolactinoma and were treated with temozolomide. One patient died in the 7th postoperative month due to pneumonia and septic shock, while another patient died in the 15th postoperative month due to a malignant prolactinoma.

### 3.5 Factors associated with the amount of resection

**Figure 3:**
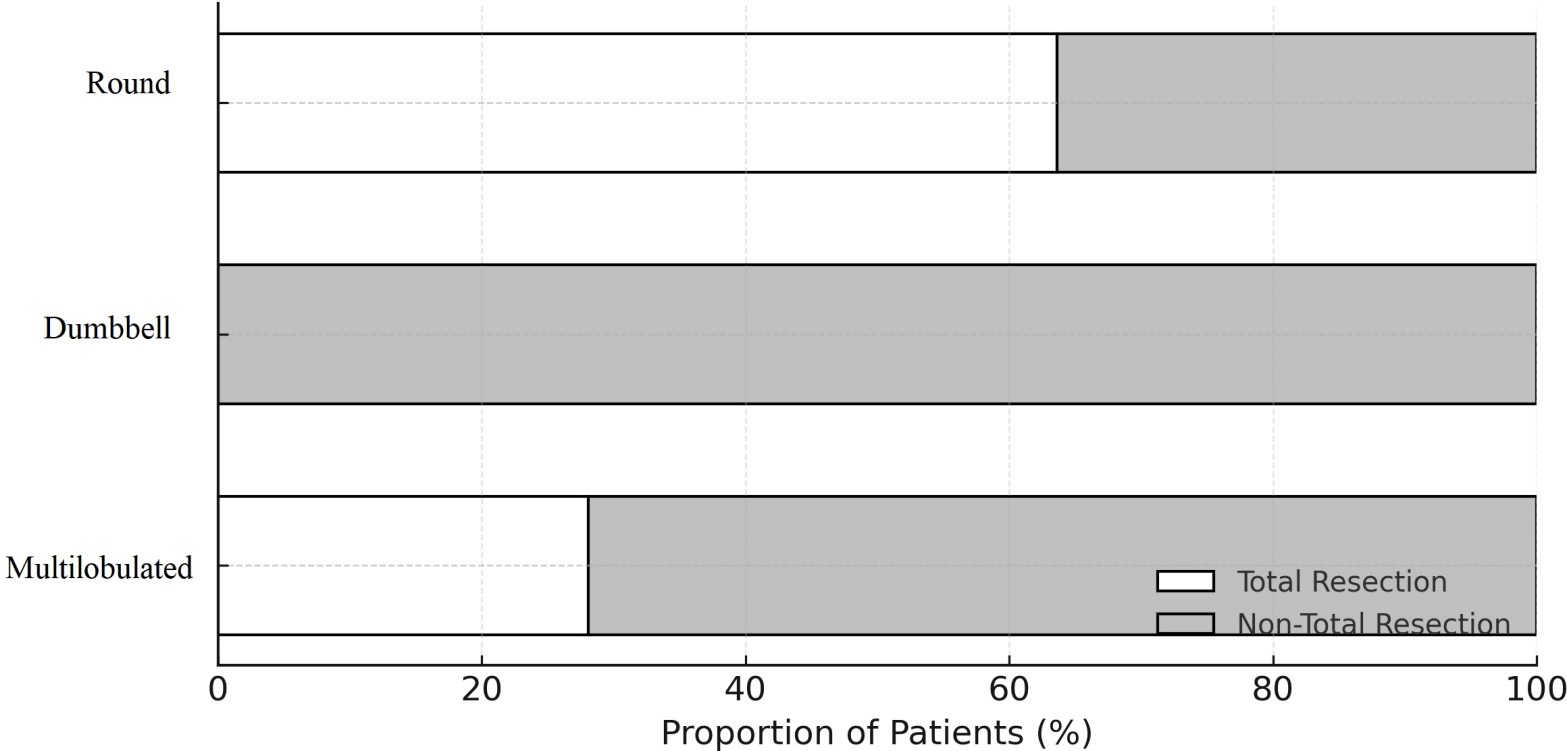
Comparison of surgical resection amounts according to adenoma shape. There was a significant difference in the amount of resection between the three groups (p = 0.003).

**Figure 4:**
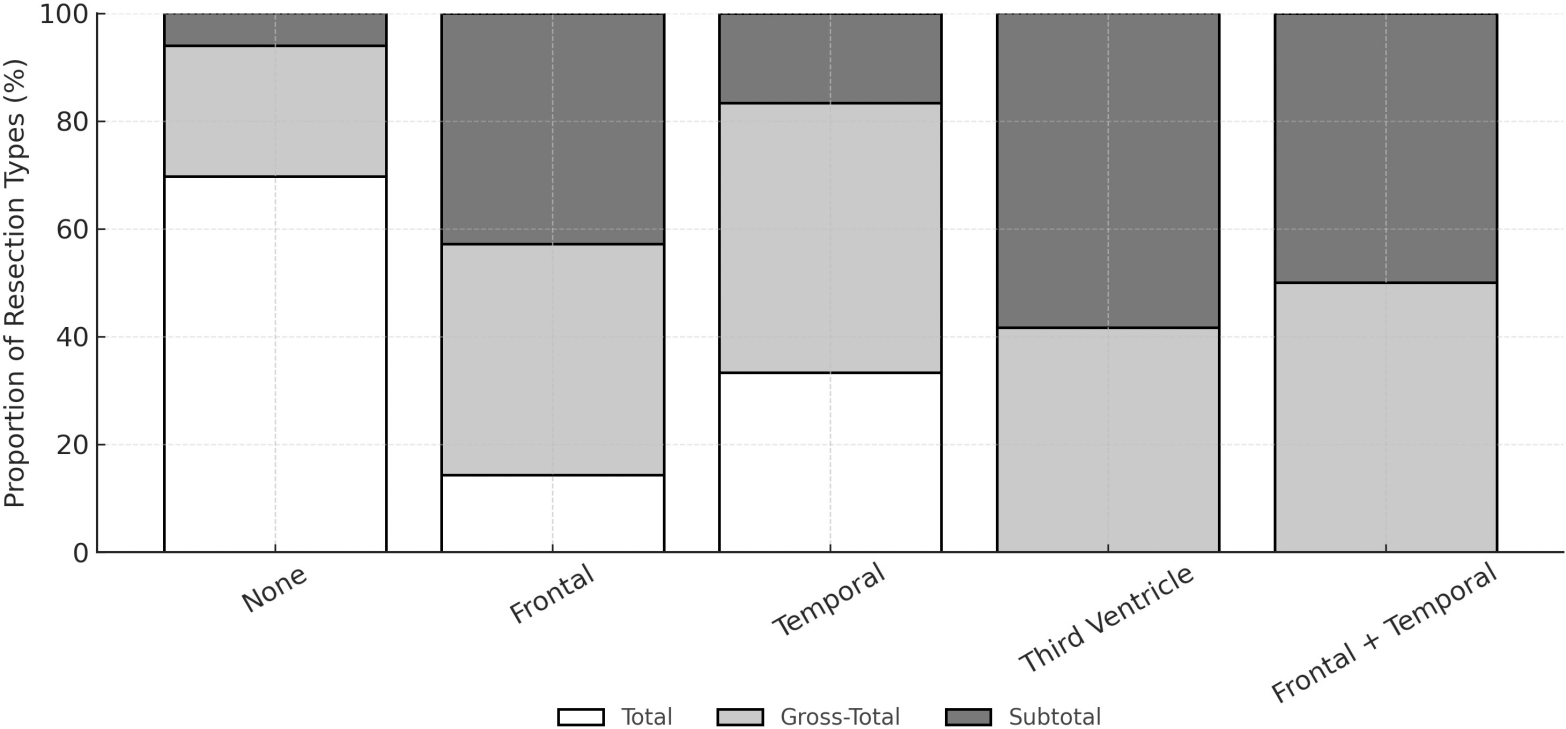
Comparison of surgical resection amounts according to the cranial extension of the adenoma. There was a significant difference in the amount of resection between the three groups (p < 0.001).

**Figure 5:**
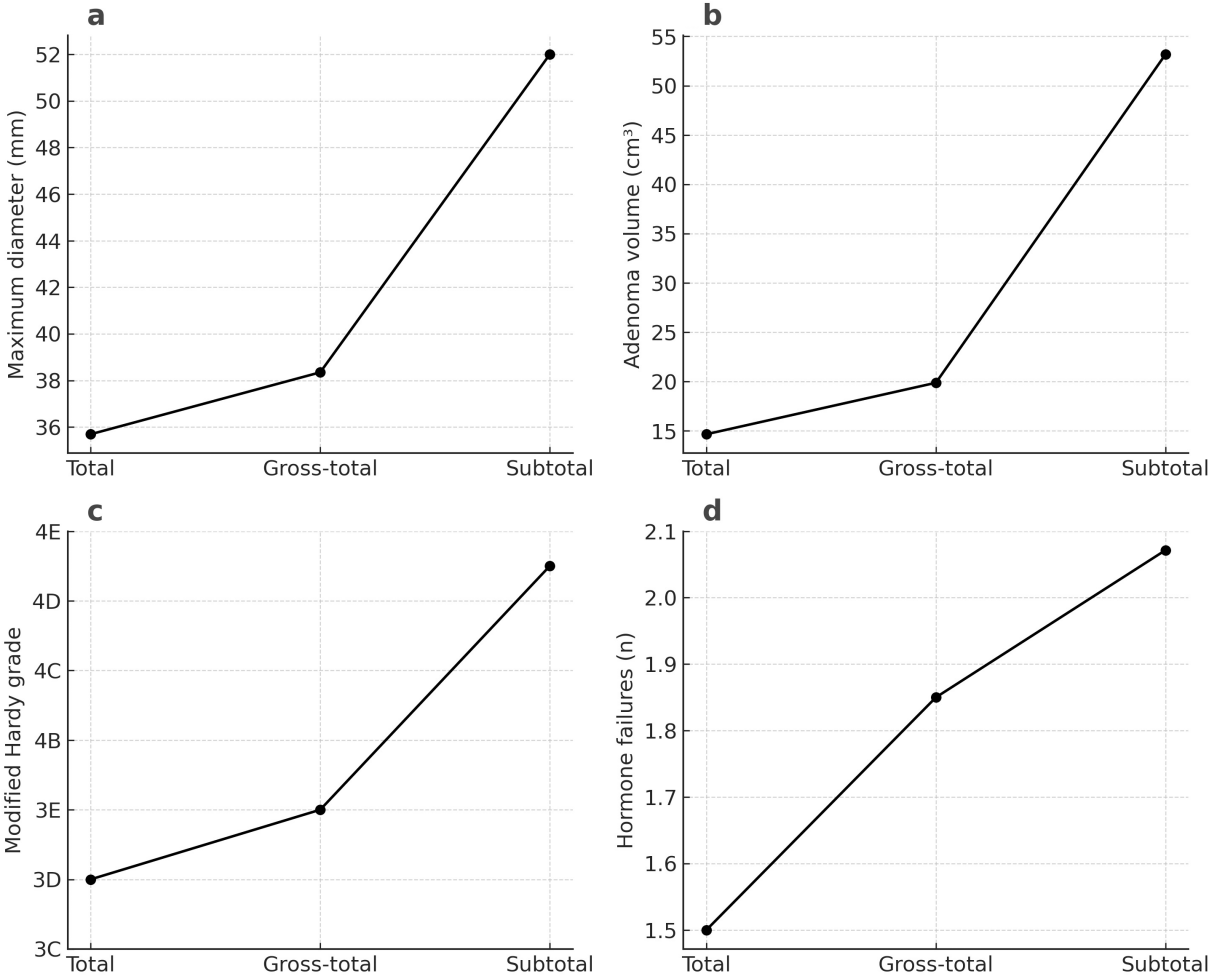
Comparison of factors related to the amount of resection between groups. **(a)** Relationship between the largest diameter of the adenoma and the amount of resection; **(b)** Relationship between adenoma volume and the amount of resection; **(c)** Relationship between Modified Hardy grade and the amount of resection; **(d)** Relationship between the mean number of missing anterior pituitary hormones and resection at admission (p < 0.05 for all).

Our observations showed an association between the shape of the adenoma and the extent of resection (p = 0.003). Round adenomas (n=14, 53.8%) had the highest rate of total resection, while multilobulated adenomas (n=9, 28.1%) had the lowest rate (**Figure 3**). Another factor influencing the extent of resection was the presence and location of the cranial extension of the adenoma (p < 0.001). While total resection was likely highest when there was no cranial extension, adenomas that extended to the third ventricle and simultaneously to the frontal and temporal lobes were associated with the inability to achieve total resection (**Figure 4**). Patients who achieved total resection had a smaller adenoma diameter (p < 0.001), a smaller adenoma volume (p < 0.001), and a lower Hardy grade (p = 0.027, **Figure 5**). There were some differences in the distribution of resection volume among the pathological subtypes of adenomas. However, comparative statistical analysis could not be performed because some functional tumor categories consisted of a small number of patients. Nevertheless, the number of resections according to pathological type is given in the **Supplementary Table**.

### 3.6 Predictive modeling for total resection

**Figure 6:**
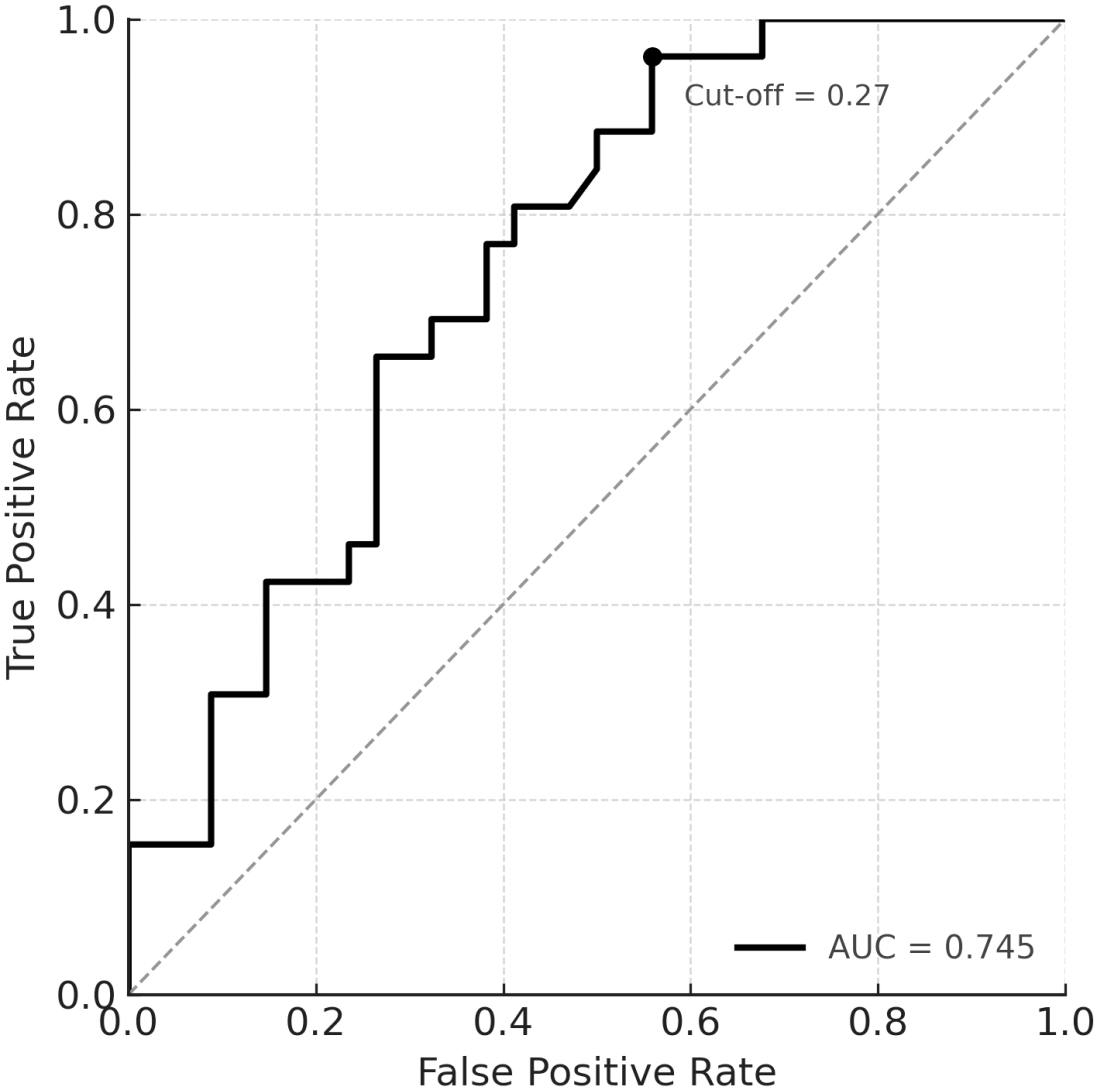
Receiver operating characteristic (ROC) curve of the multivariate logistic regression model predicting total tumor resection based on tumor volume, maximum diameter, modified Hardy grade, and the number of anterior pituitary hormone deficiencies. The area under the curve (AUC) was 0.745 (95% CI: 0.672–0.892), indicating moderate discriminative ability. The optimal probability cut-off point was 0.274, corresponding to a sensitivity of 96.2% and a specificity of 44.1%. The cut-off threshold is marked on the curve.

To investigate the ability of preoperative factors to predict total tumor resection, a multivariate logistic regression model was constructed including tumor volume (cm³), maximum tumor diameter (mm), modified Hardy grade, and the number of anterior pituitary hormone deficiencies at admission. The model demonstrated acceptable discriminatory power, with receiver operating characteristic (ROC) analysis yielding an area under the curve (AUC) of 0.745, with a 95% confidence interval (CI) of 0.672 to 0.892, calculated using a 1000-iteration bootstrap resampling method. The optimal probability cut-off value for predicting total resection, determined by the Youden index, was 0.274. At this threshold, the model achieved a sensitivity of 96.2%, specificity of 44.1%, positive predictive value (PPV) of 61.8%, and negative predictive value (NPV) of 92.3%, based on the observed sample distribution. These findings indicate that the model is highly sensitive and effective in identifying patients who are likely to undergo total resection, although its moderate specificity may result in an increased false-positive rate. The ROC curve, along with the optimal threshold point, is illustrated in **Figure 6**.

## 4 Discussion

The study aimed to comprehensively evaluate patients who underwent surgery for a giant pituitary adenoma at a tertiary pituitary center. The results showed that total or gross-total resection was achieved in most patients. Twenty percent of patients required a second endoscopic transsphenoidal procedure. In most patients, the visual impairment, the main reason for admission, improved. However, preoperative hypopituitarism persisted in the majority of patients. Remarkably, there were no cases of severe vascular nerve injury or postoperative death. The incidence of postoperative persistent diabetes insipidus was very low. Factors such as size and volume of the adenoma, presence, and location of cranial extension, advanced Hardy stage, and multilobulated structure negatively influenced resection success. Overall, the study showed that endoscopic transsphenoidal surgery is highly effective and safe in treating giant pituitary adenomas.

Although several studies have evaluated surgical outcomes in giant pituitary adenomas, our findings contribute novel insights by quantifying the impact of multilobulated tumor morphology and distinct cranial extension patterns on the extent of resection. Prior literature has acknowledged the complexity of these features, but few studies have systematically analyzed their independent predictive value. The present results highlight that tumor geometry—particularly multilobulated configuration and superior extension into the third ventricle or combined frontal/temporal compartments—represents a major determinant of resectability, often outweighing classical invasion metrics such as Knosp grade. These observations refine the anatomical understanding of giant adenomas and offer actionable parameters for preoperative planning.

The primary objectives in the surgical management of giant pituitary adenomas are to decompress the optic pathways, achieve maximal tumor removal without compromising critical neurovascular structures, and preserve or improve endocrine function. Due to the size, extension, and heterogeneous morphology of these tumors, complete resection remains a challenge. Previous studies have reported total resection rates ranging from 14.7% to 56.4%, while the combination of total and gross-total resections typically raises the overall surgical success rate to between 56% and 85% (3, 22–29). In the present series, total resection was achieved in 43.3% of cases, and when gross-total resections were included, the combined rate reached 76.7%, aligning with the upper range of previously reported outcomes. This consistency suggests that the resectability of giant adenomas is largely determined by anatomical and morphological factors rather than surgical strategy alone.

In addition to anatomical factors, several technical nuances of our surgical approach may have contributed to the favorable outcomes in this cohort. These include early central debulking to promote inferior tumor descent, wide anterior sphenoidotomies with tailored transtubercular or transplanar extensions when superior access was required, and systematic use of angled endoscopes and microinstruments to navigate multilobulated compartments. Furthermore, selective cavernous sinus exploration guided by Doppler ultrasonography was performed only when safe corridors were clearly identifiable. Consistent skull base reconstruction using a vascularized nasoseptal flap likely contributed to the very low cerebrospinal fluid leak rate. These refinements, although individually described in the literature, have rarely been evaluated together in a uniform surgical strategy and may help explain the low morbidity profile of this series.

The need for a second ETSS may arise in patients who develop recurrent symptoms due to tumor regrowth or in cases where residual tumor components, particularly those with cranial or lateral extension, are inaccessible during the initial surgery. This scenario is often encountered in tumors with aggressive behavior, such as elevated Ki-67 indices or high mitotic activity, which limit the possibility of achieving total resection in a single session (22–29). In certain cases, strategic maximal debulking in the initial procedure may facilitate the descent of residual tumor tissue into the sellar region, enabling subsequent removal via a second ETSS. This staged approach is considered a reasonable alternative to combined transcranial-transsphenoidal interventions, which carry higher morbidity. In our series, approximately 20% of patients underwent a second ETSS, consistent with prior reports and reflecting the necessity of a multimodal surgical strategy in anatomically complex or invasive tumors.

Visual impairment was the most common presenting symptom among patients with giant pituitary adenomas, typically resulting from optic chiasm compression due to suprasellar tumor extension. Interestingly, several patients who did not report subjective visual disturbances were found to have subclinical deficits on formal visual field testing, highlighting the importance of objective evaluation in all cases. Surgical decompression via ETSS led to significant improvement in visual function in the majority of patients, consistent with previous studies reporting postoperative visual field recovery in 70% to 90% of cases (22–29). In this context, restoration of visual function represents one of the most tangible and impactful benefits of surgical intervention, contributing directly to improved quality of life and functional independence.

Anterior pituitary hormone deficiencies were highly prevalent at presentation, reflecting the long-standing compressive effects of giant adenomas on the normal glandular tissue. Although surgical intervention may alleviate some of this pressure, recovery of endocrine function postoperatively is often limited. In most cases, preoperative hypopituitarism persists after tumor resection, and new hormone deficits may also arise due to intraoperative manipulation of residual pituitary tissue or vascular compromise. These findings are in line with previous studies suggesting that hormonal recovery following surgery is unpredictable and highly dependent on tumor size, duration of gland compression, preoperative hormonal reserve, and the extent of resection (30). Consequently, long-term hormone replacement remains a cornerstone of management in this patient population, regardless of surgical outcome.

Major surgical complications such as neurovascular injury or perioperative mortality were not observed in this cohort, highlighting the overall safety profile of the endoscopic transsphenoidal approach in experienced hands. Cerebrospinal fluid leakage, a commonly reported complication in the surgical treatment of giant pituitary adenomas, was effectively minimized by the consistent use of vascularized nasoseptal flaps for skull base reconstruction. This technique, now standard in high-volume centers, has been shown to significantly reduce the incidence of postoperative CSF leaks and related morbidity (31, 32). Another relevant complication, DI, occurred at a lower-than-expected rate in our series. While the literature reports a permanent DI incidence of up to 10% following giant adenoma surgery (22–29), our observed rate was considerably lower. This may be attributed to the meticulous intraoperative identification and preservation of the neurohypophysis and infundibulum during tumor dissection, which are key strategies in reducing the risk of permanent DI without compromising surgical goals.

Suprasellar and lateral cranial extensions—particularly into the frontal lobe, temporal lobe, or third ventricle—emerged as the most significant anatomical barriers to achieving complete resection in giant pituitary adenomas. These extensions often indicate a multilobulated growth pattern, which radiologically reflects complex tumor geometry and invasiveness. Such configurations hinder surgical access and visibility, even with advanced techniques. While traditional microscopic approaches offer limited maneuverability in these regions, ETSS provides enhanced illumination and panoramic visualization, facilitating access to difficult areas at the skull base (33). Nevertheless, in select cases with extensive anterior or lateral extension, complete resection may require a combined transcranial and transsphenoidal approach. ETSS remains the primary modality for initial tumor debulking in most cases, but multimodal strategies—including repeat surgery, medical therapy, and radiotherapy—are often necessary to achieve durable disease control. Importantly, maximal surgical debulking can enhance the efficacy of adjuvant treatments by improving drug penetration and reducing tumor burden (22, 25–27).

Cavernous sinus invasion, typically evaluated using the Knosp grading system, is a well-recognized factor limiting the extent of pituitary adenoma resection. In this study, nearly all patients exhibited radiological evidence of cavernous sinus involvement, yet no significant association was observed between Knosp grade and the degree of surgical resection achieved. This lack of correlation may reflect the uniformly high prevalence of advanced invasion in the study cohort, thereby reducing discriminatory power. Furthermore, recent developments in endoscopic technique, including angled visualization and the use of intraoperative Doppler probes, have expanded the ability to safely access tumor components within the cavernous compartment (34). These advancements may help explain why cavernous sinus invasion, although technically challenging, does not necessarily preclude effective tumor debulking in selected cases. However, given the critical neurovascular structures involved, radical resection in this region must always be balanced against the risk of morbidity.

In recent years, efforts to identify preoperative predictors of resection success in giant pituitary adenomas have gained momentum, aiming to support surgical planning and patient counseling. In line with this objective, we explored the combined predictive value of tumor-related factors such as volume, maximum diameter, cranial extension, and hormonal deficits. The resulting model demonstrated a clinically meaningful ability to differentiate patients with favorable surgical outcomes. These findings are consistent with previous studies emphasizing the importance of anatomical configuration and endocrine involvement as markers of surgical complexity. While imaging-based parameters like the Knosp and Hardy grades have traditionally guided expectations for resection, our results underscore the additive value of integrating volumetric and hormonal assessments. Such models may serve as useful adjuncts in stratifying surgical risk and tailoring multimodal treatment strategies, particularly in borderline resectable cases. Recent years have seen increasing interest in predictive modeling approaches aimed at improving preoperative risk stratification in pituitary disorders. Zhang et al. recently demonstrated the feasibility of federated learning–based models to predict postoperative remission in patients with acromegaly across multiple centers (35). In contrast, our model focuses on readily available anatomical variables in giant pituitary adenomas, where surgical complexity is primarily driven by tumor geometry and extension rather than biochemical remission alone. In addition, the study by Zhu et al. on suprasellar pituitary adenomas supports our observation that the pattern and direction of cranial extension play a critical role in determining surgical accessibility and the extent of resection (36).

Residual tumor apoplexy represents a clinically relevant postoperative risk in giant adenomas, particularly when substantial suprasellar or multilobulated remnants persist. Although no case of postoperative apoplexy occurred in our cohort, this complication warrants explicit consideration given its potential for rapid visual deterioration. Romano et al. have highlighted the importance of early recognition and prompt surgical intervention in such cases (37). In our center, patients with significant residual tumor undergo structured early imaging at 3–4 weeks, formal endocrine reassessment, and expedited evaluation of new-onset headaches or visual complaints. These measures may have contributed to the absence of apoplexy in our series and underscore the value of proactive surveillance in high-risk patients.

Our findings also align with and extend the observations of Chibbaro et al., who emphasized the technical challenges and heterogeneity of outcomes in a large prospective series of giant adenomas (38). However, the present study expands on their work by quantitatively assessing the independent influence of multilobulated morphology and compartment-specific cranial extension patterns—variables not separately analyzed in most previous studies. This differentiation provides a more granular, anatomy-based understanding of resectability and may help refine preoperative decision-making.

Descriptively, the distribution of gross-total and subtotal resection varied across pathological subtypes; however, the small number of patients in several functional adenoma categories limited meaningful comparative analysis. Overall, no consistent pattern suggesting superior or inferior resectability based solely on pathological subtype was observed, with anatomical factors remaining the primary determinants of surgical outcome.

Several limitations should be considered when interpreting the findings of this study. First, the retrospective and single-center design may limit the generalizability of the results. In addition, while detailed morphologic and surgical data were analyzed, the biological behavior of giant pituitary adenomas—such as proliferative index, invasiveness, and molecular markers—was not systematically evaluated and may further inform prognosis in future studies. The nearly universal presence of cavernous sinus invasion in the cohort may also have limited the assessment of this factor’s predictive value. Another limitation of this study is that the extent of resection was assessed based on early postoperative imaging. Although follow-up data, including tumor recurrence and the need for repeat surgery or adjuvant therapy, were available and are reported, small residual tumor remnants—particularly along the cavernous sinus walls—may not be detectable on early MRI. Despite these limitations, the study provides a comprehensive clinical and surgical profile of a challenging tumor entity and contributes to the growing body of literature supporting ETSS as a primary modality in the management of giant pituitary adenomas. By identifying key anatomical and structural predictors of resection success, the findings may assist in surgical planning and patient counseling in similar high-complexity cases.

## 5 Conclusion

Giant pituitary adenomas pose significant therapeutic challenges due to their size, complex morphology, and frequent extension into critical neuroanatomical structures. ETSS remains the cornerstone of initial treatment, offering effective tumor debulking with favorable visual and neurological outcomes. Advances in endoscopic techniques and accumulated surgical experience have enabled more extensive resections to be performed with low rates of morbidity and mortality. Nonetheless, complete resection is often limited by tumor volume, maximum diameter, multilobulated architecture, and cranial extension patterns. The present study highlights the independent predictive value of these anatomical and morphological factors, demonstrating that multilobulated configuration and superior/lateral extension trajectories are among the strongest determinants of resectability in giant adenomas. By integrating volumetric, anatomical, and endocrine variables into a multivariate model, our findings provide a practical preoperative tool to estimate the likelihood of total resection and guide individualized surgical planning. This anatomy-informed framework may help identify patients who would benefit from early consideration of staged procedures or adjunctive therapies. In cases where total resection is not feasible, multimodal strategies—including repeat ETSS, medical therapy, and radiotherapy—allow for adequate long-term disease control in most patients.

## Data Availability

The raw data supporting the conclusions of this article will be made available by the authors, without undue reservation.

## References

[1] Di Ieva A Rotondo F Syro LV Cusimano MD Kovacs K . Aggressive pituitary adenomas–diagnosis and emerging treatments. Nat Rev Endocrinol. (2014) 10:423–35. doi: 10.1038/nrendo.2014.64, PMID: 24821329

[2] Chacko G Chacko AG Lombardero M Mani S Seshadri MS Kovacs K . Clinicopathologic correlates of giant pituitary adenomas. J Clin Neurosci. (2009) 16:660–5. doi: 10.1016/j.jocn.2008.08.018, PMID: 19285407

[3] Goel A Nadkarni T Muzumdar D Desai K Phalke U Sharma P . Giant pituitary tumors: a study based on surgical treatment of 118 cases. Surg Neurol. (2004) 61:436–46. doi: 10.1016/j.surneu.2003.08.036, PMID: 15120215

[4] Vargas G Gonzalez B Ramirez C Ferreira A Espinosa E Mendoza V . Clinical characteristics and treatment outcome of 485 patients with nonfunctioning pituitary macroadenomas. Int J Endocrinol. (2015) 2015:756069. doi: 10.1155/2015/756069, PMID: 25737722 PMC4337176

[5] Nimsky C von Keller B Ganslandt O Fahlbusch R . Intraoperative high-field magnetic resonance imaging in transsphenoidal surgery of hormonally inactive pituitary macroadenomas. Neurosurgery. (2006) 59:105–14. doi: 10.1227/01.NEU.0000219198.38423.1E, PMID: 16823306

[6] Casanueva FF Barkan AL Buchfelder M Klibanski A Laws ER Loeffler JS . Criteria for the definition of pituitary tumor centers of excellence (PTCOE): A pituitary society statement. Pituitary. (2017) 20:489–98. doi: 10.1007/s11102-017-0838-2, PMID: 28884415 PMC5606938

[7] Jho HD Carrau RL . Endoscopic endonasal transsphenoidal surgery: experience with 50 patients. J Neurosurg. (1997) 87:44–51. doi: 10.3171/jns.1997.87.1.0044, PMID: 9202264

[8] Cappabianca P Alfieri A de Divitiis E . Endoscopic endonasal transsphenoidal approach to the sella: towards functional endoscopic pituitary surgery (FEPS). Minim Invasive Neurosurg. (1998) 41:66–73. doi: 10.1055/s-2008-1052019, PMID: 9651913

[9] Penn DL Burke WT Laws ER . Management of non-functioning pituitary adenomas: surgery. Pituitary. (2018) 21:145–53. doi: 10.1007/s11102-017-0854-2, PMID: 29280026

[10] Iglesias P Rodríguez Berrocal V Díez JJ . Giant pituitary adenoma: histological types, clinical features and therapeutic approaches. Endocrine. (2018) 61:407–21. doi: 10.1007/s12020-018-1645-x, PMID: 29909598

[11] Melmed S Casanueva FF Hoffman AR Kleinberg DL Montori VM Schlechte JA . Diagnosis and treatment of hyperprolactinemia: an Endocrine Society clinical practice guideline. J Clin Endocrinol Metab. (2011) 96:273–88. doi: 10.1210/jc.2010-1692, PMID: 21296991

[12] Katznelson L Laws ER Jr Melmed S Molitch ME Murad MH Utz A . Acromegaly: an endocrine society clinical practice guideline. J Clin Endocrinol Metab. (2014) 99:3933–51. doi: 10.1210/jc.2014-2700, PMID: 25356808

[13] Nieman LK Biller BM Findling JW Newell-Price J Savage MO Stewart PM . The diagnosis of Cushing's syndrome: an Endocrine Society Clinical Practice Guideline. J Clin Endocrinol Metab. (2008) 93:1526–40. doi: 10.1210/jc.2008-0125, PMID: 18334580 PMC2386281

[14] Nieman LK Biller BM Findling JW Murad MH Newell-Price J Savage MO . Treatment of cushing's syndrome: an endocrine society clinical practice guideline. J Clin Endocrinol Metab. (2015) 100:2807–31. doi: 10.1210/jc.2015-1818, PMID: 26222757 PMC4525003

[15] Ding D Starke RM Sheehan JP . Treatment paradigms for pituitary adenomas: defining the roles of radiosurgery and radiation therapy. J Neurooncol. (2014) 117:445–57. doi: 10.1007/s11060-013-1262-8, PMID: 24122025

[16] Moisi M Cruz AS Benkers T Rostad S Broyles FB Yuen K . Treatment of aggressive prolactin-secreting pituitary adenomas with adjuvant temozolomide chemotherapy: A review. Cureus. (2016) 8:e658. doi: 10.7759/cureus.658, PMID: 27489751 PMC4963231

[17] Dickstein G Shechner C . Low dose ACTH test–a word of caution to the word of caution: when and how to use it. J Clin Endocrinol Metab. (1997) 82:322. doi: 10.1210/jcem.82.1.3704-1, PMID: 8989282

[18] Bidlingmaier M Friedrich N Emeny RT Spranger J Wolthers OD Roswall J . Reference intervals for insulin-like growth factor-1 (igf-i) from birth to senescence: results from a multicenter study using a new automated chemiluminescence IGF-I immunoassay conforming to recent international recommendations. J Clin Endocrinol Metab. (2014) 99:1712–21. doi: 10.1210/jc.2013-3059, PMID: 24606072

[19] Fleseriu M Hashim IA Karavitaki N Melmed S Murad MH Salvatori R . Hormonal replacement in hypopituitarism in adults: an endocrine society clinical practice guideline. J Clin Endocrinol Metab. (2016) 101:3888–921. doi: 10.1210/jc.2016-2118, PMID: 27736313

[20] Wilson CB . A decade of pituitary microsurgery. The Herbert Olivecrona lecture. J Neurosurg. (1984) 61:814–33. doi: 10.3171/jns.1984.61.5.0814, PMID: 6092567

[21] Patel SK Husain Q Eloy JA Couldwell WT Liu JK . Norman Dott, Gerard Guiot, and Jules Hardy: key players in the resurrection and preservation of transsphenoidal surgery. Neurosurg Focus. (2012) 33:E6. doi: 10.3171/2012.6.FOCUS12125, PMID: 22853837

[22] Koutourousiou M Gardner PA Fernandez-Miranda JC Paluzzi A Wang EW Snyderman CH . Endoscopic endonasal surgery for giant pituitary adenomas: advantages and limitations. J Neurosurg. (2013) 118:621–31. doi: 10.3171/2012.11.JNS121190, PMID: 23289816

[23] Juraschka K Khan OH Godoy BL Monsalves E Kilian A Krischek B . Endoscopic endonasal transsphenoidal approach to large and giant pituitary adenomas: institutional experience and predictors of extent of resection. J Neurosurg. (2014) 121:75–83. doi: 10.3171/2014.3.JNS131679, PMID: 24785323

[24] de Paiva Neto MA Vandergrift A Fatemi N Gorgulho AA Desalles AA Cohan P . Endonasal transsphenoidal surgery and multimodality treatment for giant pituitary adenomas. Clin Endocrinol (Oxf). (2010) 72:512–9. doi: 10.1111/j.1365-2265.2009.03665.x, PMID: 19555365

[25] Mortini P Barzaghi R Losa M Boari N Giovanelli M . Surgical treatment of giant pituitary adenomas: strategies and results in a series of 95 consecutive patients. Neurosurgery. (2007) 60:993–1004. doi: 10.1227/01.NEU.0000255459.14764.BA, PMID: 17538372

[26] Chabot JD Chakraborty S Imbarrato G Dehdashti AR . Evaluation of outcomes after endoscopic endonasal surgery for large and giant pituitary macroadenoma: A retrospective review of 39 consecutive patients. World Neurosurg. (2015) 84:978–88. doi: 10.1016/j.wneu.2015.06.007, PMID: 26074433

[27] Rahimli T Hidayetov T Yusifli Z Memmedzade H Rajabov T Aghayev K . Endoscopic endonasal approach to giant pituitary adenomas: surgical outcomes and review of the literature. World Neurosurg. (2021) 149:e1043–55. doi: 10.1016/j.wneu.2021.01.019, PMID: 33524611

[28] Gondim JA Almeida JP Albuquerque LA Gomes EF Schops M . Giant pituitary adenomas: surgical outcomes of 50 cases operated on by the endonasal endoscopic approach. World Neurosurg. (2014) 82:e281–90. doi: 10.1016/j.wneu.2013.08.028, PMID: 23994073

[29] Sinha S Sharma BS . Giant pituitary adenomas–an enigma revisited. Microsurgical treatment strategies and outcome in a series of 250 patients. Br J Neurosurg. (2010) 24:31–9. doi: 10.3109/02688690903370305, PMID: 20158350

[30] Barber TM Kyrou I Kaltsas G Grossman AB Randeva HS Weickert MO . Mechanisms of central hypogonadism. Int J Mol Sci. (2021) 22:8217. doi: 10.3390/ijms22158217, PMID: 34360982 PMC8348115

[31] Kassam AB Thomas A Carrau RL Snyderman CH Vescan A Prevedello D . Endoscopic reconstruction of the cranial base using a pedicled nasoseptal flap. Neurosurgery. (2008) 63:ONS44–53. doi: 10.1227/01.neu.0000297074.13423.f5, PMID: 18728603

[32] Zanation AM Carrau RL Snyderman CH Germanwala AV Gardner PA Prevedello DM . Nasoseptal flap reconstruction of high flow intraoperative cerebral spinal fluid leaks during endoscopic skull base surgery. Am J Rhinol Allergy. (2009) 23:518–21. doi: 10.2500/ajra.2009.23.3378, PMID: 19807986

[33] Castelnuovo P Dallan I Battaglia P Bignami M . Endoscopic endonasal skull base surgery: past, present and future. Eur Arch Otorhinolaryngol. (2010) 267:649–63. doi: 10.1007/s00405-009-1196-0, PMID: 20063006

[34] Dolati P Eichberg D Golby A Zamani A Laws E . Multimodal navigation in endoscopic transsphenoidal resection of pituitary tumors using image-based vascular and cranial nerve segmentation: A prospective validation study. World Neurosurg. (2016) 95:406–13. doi: 10.1016/j.wneu.2016.06.008, PMID: 27302558 PMC5143211

[35] Zhang W Wu X Wang H Wu R Deng C Xu Q . Federated learning for predicting postoperative remission of patients with acromegaly: A multicentered study. World Neurosurg. (2025) 193:1036–46. doi: 10.1016/j.wneu.2024.10.091, PMID: 39486577

[36] Zhu J Wang Z Zhang Y Liu J Li X Deng K . Suprasellar pituitary adenomas: a 10-year experience in a single tertiary medical center and a literature review. Pituitary. (2020) 23:367–80. doi: 10.1007/s11102-020-01043-1, PMID: 32378170

[37] Romano A Ganau M Zaed I Scibilia A Oretti G Chibbaro S . Primary endoscopic management of apoplexy in a giant pituitary adenoma. World Neurosurg. (2020) 142:312–3. doi: 10.1016/j.wneu.2020.07.059, PMID: 32702495

[38] Chibbaro S Signorelli F Milani D Cebula H Scibilia A Bozzi MT . Primary endoscopic endonasal management of giant pituitary adenomas: outcome and pitfalls from a large prospective multicenter experience. Cancers (Basel). (2021) 13:3603. doi: 10.3390/cancers13143603, PMID: 34298816 PMC8304085

