# Supplementary material for: Predicting resection success in giant pituitary adenomas: morphologic determinants and a preoperative multivariate model
Source: Front Endocrinol (Lausanne). 2026 Jan 30;17:1759071. doi: 10.3389/fendo.2026.1759071 (PMC12900767; doi:10.3389/fendo.2026.1759071)
Supplement: Supplementary file 1 [file Table1.docx]

**Supplementary Table.** Comparison of resection amounts according to tumor pathological type.

| **Pathological Type** | **Resection Amount** | | | **Number of Patients** |
| --- | --- | --- | --- | --- |
|  | **Total** | **Gross-total** | **Subtotal** |  |
| Null-cell adenoma, n | 1 | 1 | 1 | 3 |
| Gonadotropin adenoma, n | 15 | 9 | 8 | 32 |
| Prolactin-secreting adenoma, n | 4 | 3 | 4 | 11 |
| Growth hormone-secreting adenomas, n | 2 | 0 | 0 | 2 |
| ACTH-secreting adenoma, n | 0 | 2 | 0 | 2 |
| Mixt-plurihormonal adenoma, n | 4 | 5 | 1 | 10 |
| Total, n | 26 | 20 | 14 | 60 |
